# ECMO Before Heart Transplantation: Early Implantation and Optimized Assistance with the Eurosets ECMOLIFE System and Landing Advance—A Case Report

**DOI:** 10.3390/reports9020105

**Published:** 2026-03-28

**Authors:** Giuseppe Santarpino, Alessandro Fiorentino, Federico Cucci, Veronica D’Anna, Giuseppe Speziale

**Affiliations:** 1 Department of Experimental and Clinical Medicine, Magna Graecia University, 88100 Catanzaro, Italy; 2 Department of Cardiac Surgery, Città di Lecce Hospital, 73100 Lecce, Italy; afiorentino@gvmnet.it (A.F.); fcucci@gvmnet.it (F.C.); veronicadanna21@gmail.com (V.D.); gspeziale@gvmnet.it (G.S.); 3 PhD Program “Life Sciences and Technologies”, Magna Graecia University, 88100 Catanzaro, Italy; 4 Department of Cardiac Surgery, Santa Maria Hospital GVM Care & Research, 70124 Bari, Italy; 5 Department of Health and Life Sciences, European University of Rome, 00163 Rome, Italy

**Keywords:** extracorporeal membrane oxygenation, cardiogenic shock, heart transplantation, veno-arterial ECMO, case report

## Abstract

**Background:** Extracorporeal membrane oxygenation (ECMO) is commonly used for temporary support in patients with severe cardiogenic shock and may serve as a bridge to heart transplantation. In recent years, outcomes have improved with better timing, patient management and advances in ECMO technology. **Case presentation**: We describe the case of a 61-year-old man who developed refractory cardiogenic shock after an extensive acute myocardial infarction complicated by recurrent ventricular arrhythmias. After an initial period of stabilization following complex percutaneous coronary intervention, the patient suddenly deteriorated with acute pulmonary edema and severe hypoxemia. A peripheral femoro-femoral veno-arterial ECMO with distal limb perfusion was promptly implanted using the ECMOLIFE system and the Landing Advance system (Eurosets s.r.l., Medolla, MO, Italy) to stabilize the patient and enable continuous monitoring. Due to severe left ventricular distension, surgical left ventricular venting was performed through a minimally invasive approach. ECMO support allowed rapid hemodynamic stabilization without major complications. During ECMO support, the patient remained stable and after less than 48 h a suitable donor heart became available. The patient was safely transferred to a transplant center while on ECMO and successfully underwent heart transplantation. **Conclusions**: This case shows that early ECMO implantation, combined with appropriate ventricular unloading and careful management with an advanced monitoring system, can be an optimal support as a bridge to heart transplantation. Limiting the duration of ECMO support and ensuring timely referral to a transplant center may improve outcomes in patients with refractory cardiogenic shock.

## 1. Introduction and Clinical Significance

Extracorporeal membrane oxygenation (ECMO) is increasingly utilized as a bridge to heart transplantation for patients in refractory cardiogenic shock, reflecting both advances in technology and changes in organ allocation policy that prioritize these critically ill candidates [1,2,3,4]. Historically, ECMO-bridged patients were associated with poor post-transplant outcomes and high complication rates, including increased early mortality, renal failure, and neurologic events [2,5,6]. However, contemporary data suggest that outcomes have improved with evolving management strategies and allocation systems.

Recent changes in the United States heart allocation policy have resulted in shorter waitlist times and increased likelihood of transplantation for ECMO-supported patients [1,2,3]. For example, median waitlist times for ECMO-bridged candidates decreased from 11 to 5 days after the 2018 policy change, with a corresponding reduction in waitlist death or deterioration (subdistribution hazard ratio 0.45, 95% confidence interval (CI) 0.30–0.68) [1]. Post-transplant survival has also improved: 6-month survival rates increased from 74.6% to 90.6% in the new allocation era, and 1-year survival rates for ECMO-bridged patients are now comparable to those of non-ECMO recipients in selected cohorts [1,2,3,4].

The timing of ECMO support relative to transplantation is a critical determinant of outcomes.

Here, we present a case of a patient supported for <48 h with ECMO before transplantation and a review of the literature focusing on the relationship between the time of ECMO support pre-transplant and the outcome following cardiac transplantation.

## 2. Case Presentation

On Day 0, a 61-year-old man with hypertension, type 2 diabetes mellitus, and dyslipidemia, who had already undergone percutaneous transluminal coronary angioplasty (PTCA) with drug-eluting stent (DES) on the proximal and circumflex IV in 2022 (ejection fraction (EF) then ~50% with apical/septal hypokinesia), called the 118-emergency system due to chest pain and dyspnea. On arrival at the hospital, he was hypotensive (blood pressure (BP) 80/60 mmHg), hypoxemic (SpO_2_ 80% on air) and in pulmonary subedema class IV according to the Killip classification system; the ECG showed waveform changes compatible with anterolateral ST-Elevation myocardial infarction (STEMI), while transthoracic echocardiography revealed EF 20–25% and apical and interventricular septal akinesia.

Urgent coronary angiography revealed acute thrombosis of the distal segment of the common trunk (CT) with ostial involvement of the left anterior descending coronary artery (LAD) and circumflex coronary artery (CX); the right coronary artery was ectatic without critical stenosis. Complex percutaneous coronary intervention (PCI) was then performed on the CT–LAD–CX bifurcation with the DK-Crush technique: DES Resolute Onyx 3.5 × 15 mm towards CX, mesh crushing, DES 3.5 × 18 mm from trunk to LAD, POT 4.0 mm, kissing balloons 3.5/3.5 mm and further DES 4.0 × 12 mm on proximal CT, with a TIMI 3 result. During the coronary procedure, episodes of ventricular fibrillation occurred, and were treated with shock and respiratory failure requiring orotracheal intubation. Antithrombotic therapy included heparin and intravenous cangrelor; for the anti-P2Y12 drug, prasugrel was chosen (60 mg load, then 10 mg/day) in combination with acetylsalicylic acid (ASA).

Post-coronary angioplasty, the patient was admitted to the intensive care unit (ICU) on mechanical ventilation; episodes of sustained ventricular tachycardia occurred, and low-dose dobutamine was started. Hs-troponin showed a curve consistent with extensive infarction (336,705 ng/L → peak 1,918,200 ng/L within the first 24 h → 64,885 ng/L over the following 48 h). The transesophageal echocardiogram during the early hospital course showed an EF of ~25% with anterolateral hypokinesia and preserved right ventricular function.

On Day 2, with blood gas analysis within normal limits, the patient was extubated with good clinical recovery. On the morning of Day 3, the patient was alert, eupneic, and more stable (BP 100/60 mmHg), with transfer to the ward considered.

However, later the same day, acute pulmonary edema associated with worsening of left ventricular dysfunction developed with tachycardia ~140 bpm treated with Esmolol in continuous intravenous infusion (50 mgc/Kg/min) to slow down and control the heart rhythm and severe hypoxemia, requiring sedation, orotracheal reintubation, and blood transfusion.

Heart failure with sudden loss of contractile force and inability of the ventricles to pump blood effectively, likely caused an increase in pressure in the pulmonary capillaries and the consequent passage of fluid into the alveoli with acute pulmonary edema.

**Figure 1:**
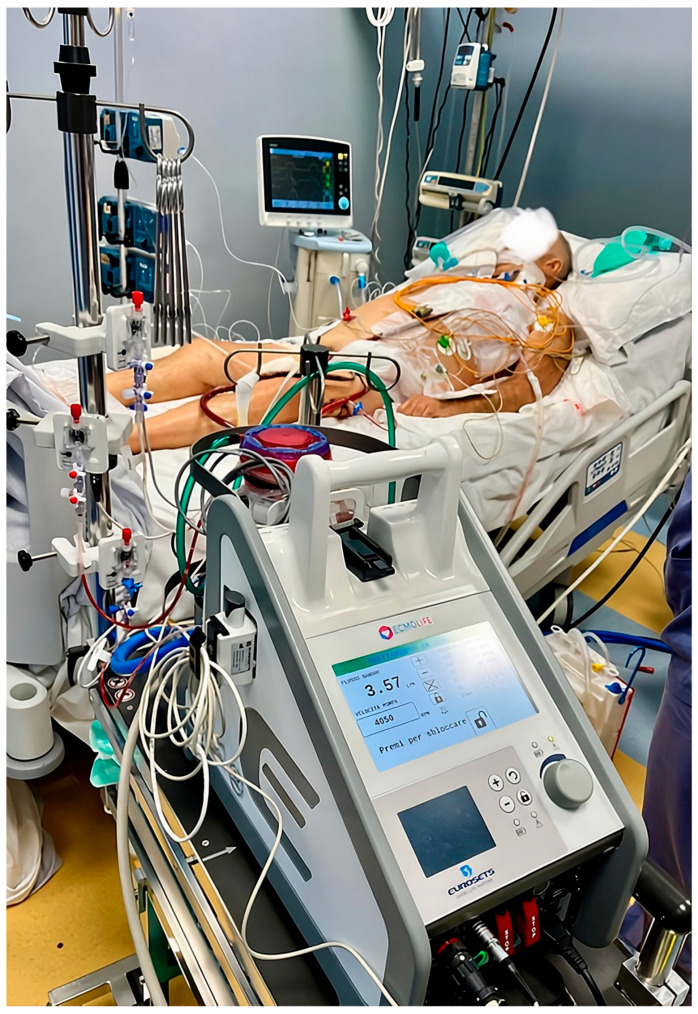
Intensive care unit box; the patient is monitored, sedated, intubated, and receiving cardiopulmonary and pharmacological support.

The patient underwent refractory cardiogenic shock that was not responsive to maximal medical therapy, for which a left peripheral femoro-femoral veno-arterial ECMO was placed using a 19 Fr arterial cannula and a 22 Fr venous cannula, along with a 9 Fr distal perfusion cannula to ensure adequate lower limb perfusion shunt (Figure 1).

Given the need for rapid hemodynamic stabilization and the potential requirement for inter-hospital transfer, veno-arterial extracorporeal membrane oxygenation was provided using the ECMOLIFE system (Eurosets s.r.l., Medolla, MO, Italy), equipped with a fully magnetic-levitation centrifugal pump, which minimizes friction, shear stress and blood stagnation, thereby reducing hemolysis. The extracorporeal circuit features a phosphorylcholine (PC) coating aimed at improving hemocompatibility and reducing platelet activation and consumption. The system is validated for continuous use for up to 30 days, allowing safe support even in cases requiring prolonged extracorporeal assistance. To enhance safety, particularly during patient transport, the ECMOLIFE platform includes an integrated back-up system, ensuring immediate pump replacement in case of device failure, without the need for manual pump operation. In addition, the ECMOLIFE system is equipped with 5 non-invasive sensors that allow continuous real-time monitoring of 11 parameters: blood flow, pump speed, drainage pressure, pre-oxygenator pressure, post-oxygenator pressure, oxygenator pressure drop, oxygen venous saturation, hemoglobin, blood venous temperature, and two air bubbles detectors. The circuit can be connected to the Landing Advance monitoring system (20 parameters in real time) for advanced hemodynamic and respiratory assessment during ECMO support and inter-hospital transfer (see Supplementary Materials File S1). Thanks to the oxygenator’s dedicated capnometer and arterial/venous probes that can be connected to any circuit, the Landing Advance monitor provides continuous measurement of the artificial lung’s contribution, along with hemoglobin, arterial, and venous oxygen saturation.

In addition, the Landing Advance provides hemodynamic data, including flow, pre-oxygenator, and post-oxygenator pressure. Of particular note is the measurement of drainage pressure; monitoring this is particularly useful for preventing hemolysis.

**Figure 2:**
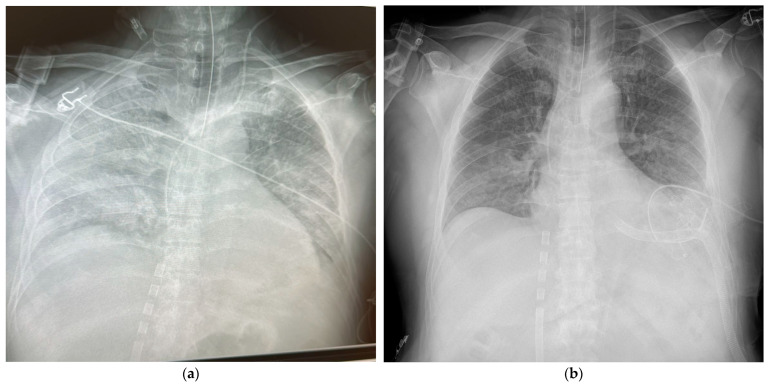
Chest X-ray. (**a**): The patient is intubated, with ECG electrodes in situ; a venous cannula correctly positioned in the right atrium; pulmonary parenchymal edema and a cardiac silhouette with dilated chambers. (**b**) The patient is intubated, with ECG electrodes in situ; a venous cannula positioned in the right atrium; decongested pulmonary parenchyma; a left ventricular cannula used as a venting system via left minithoracotomy transcostal access and well-drained cardiac chambers.

The Transplant Center was then contacted for prompt consultation. In the early afternoon of the same day, the echocardiogram showed marked distension and severe dysfunction of the left ventricle (EF ~5%) with intracavitary “smoke” effect and moderate–severe mitral regurgitation, and important indication for ventricular unloading (Figure 2a). Under general anesthesia, the intubated patient underwent apical venting of the left ventricle through a left lateral minithoracotomy, with an apical cannula connected to the ECMO venous drainage and dedicated pleural drainage (Figure 2b).

Anticoagulation was performed with an activated clotting time (ACT) target of 220–250 s. In the evening, a decrease in ECMO flow rates with hypotension was noted, which was resolved with red blood cell/albumin transfusions and vasopressor support; transesophageal echocardiography confirmed adequate biventricular drainage. Surgical revision of the femoral accesses and thoracotomy revealed no active bleeding, and blood from the drains was reinfused.

Within the first 24 h after ECMO initiation, the patient’s condition was stabilized in the critical condition: sedation was ongoing, protective ventilation was maintained, the limbs were warm and well-perfused, and the surgical accesses were dry; a chest X-ray showed progressively improved parenchymal diaphaneity (Figure 2a,b).

**Figure 3:**
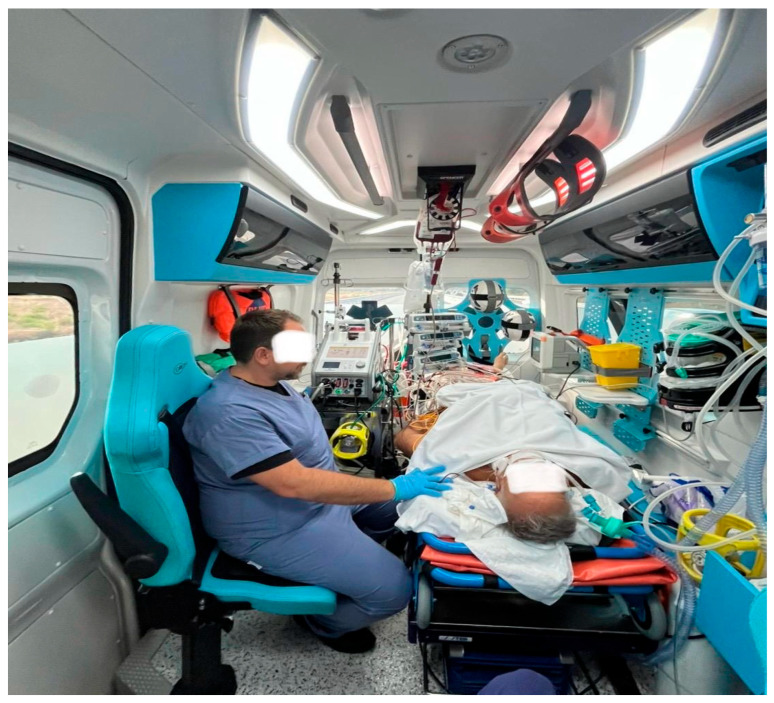
The patient was safely transported to the transplant center under stable veno-arterial ECMO support while being continuously monitored, sedated, intubated, and supported with cardiopulmonary and pharmacological therapy.

**Figure 4:**
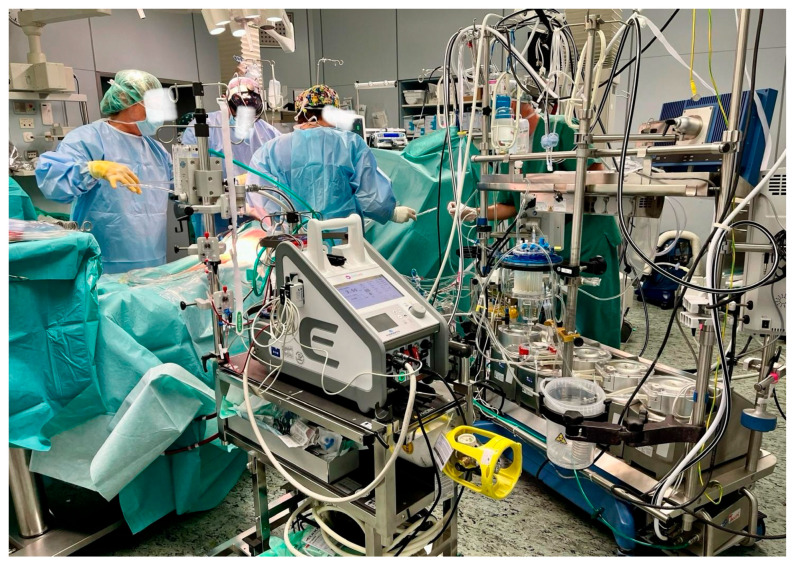
Operating room at the transplant center: upon arrival, veno-arterial ECMO support was converted to standard extracorporeal circulation (cardiopulmonary bypass) during the preoperative phase prior to heart transplantation.

Approximately 48 h after ECMO initiation, colleagues at the Bari Polyclinic Transplant Center contacted us to inform us that their team was on their way to perform a heart explant from a donor compatible with the patient admitted to our ICU. Within a few hours, after activating the transport team, organizing the ambulance, emergency supplies, and backup systems, the patient, who was intubated and on veno-arterial extracorporeal membrane oxygenation (VA ECMO) but in stable hemodynamic condition, was transferred to the Bari Polyclinic Transplant Center for the heart transplant (Figure 3). At the Bari Polyclinic, they directed us directly to the operating room, where the patient, supported by VA ECMO, was switched to conventional extracorporeal circulation (Figure 4) and prepared for the heart transplant surgery.

## 3. Discussion

ECMO has become an established first-line advanced cardiopulmonary support strategy in patients with severe cardiac and/or respiratory failure, particularly when used as a bridge to recovery, decision, or transplantation. Its rapid deployment, including bedside implantation in emergency settings, makes it a valuable option in critically ill patients. Over time, improvements in device technology, increasing clinical expertise, and broader recognition of its indications have contributed to the wider adoption of this technique. Patients transplanted directly from ECMO can achieve survival rates similar to non-ECMO recipients, provided they lack major risk factors such as mechanical ventilation or renal insufficiency [6,7,8,9]. In a large single-center study, direct transplantation from ECMO yielded 1-year survival rates of 85.5%, which were not significantly different from those of non-ECMO patients [7]. Risk stratification demonstrates that the presence of mechanical ventilation and renal insufficiency markedly increases post-transplant mortality; patients with both risk factors have 1-year survival as low as 12.5%, compared to 71.4% in those without [6,8,9]. Conversely, non-intubated ECMO patients have superior peri-transplant and long-term outcomes, with 1-year survival of 91.2% versus 85.7% in those requiring mechanical ventilation [9]. Moreover, timing of ECMO initiation appears to be a critical determinant of outcome. Recent large-scale registry data have shown that delays in ECMO initiation are associated with a progressive increase in in-hospital mortality, supporting the concept that early implementation, before the onset of irreversible organ dysfunction, may improve survival [10].

Bridging ECMO patients to a left ventricular assist device (LVAD) prior to transplantation may further improve outcomes. Patients who undergo LVAD implantation before transplant have significantly better 1-year survival compared to those transplanted directly from ECMO [11]. This suggests that, when feasible, transitioning to durable mechanical support may be advantageous, especially in patients with modifiable risk factors.

Major risk factors that worsen post-transplant outcomes in ECMO-bridged patients include mechanical ventilation, renal insufficiency, and the need for dialysis [6,8,9]. These factors should be carefully considered in relation to candidate selection and the timing of transplantation. Non-intubated ECMO patients, who are not mechanically ventilated at the time of transplant, consistently demonstrate lower rates of post-transplant stroke, dialysis, and shorter hospital stays [9].

Common complications in ECMO-bridged heart transplant recipients include stroke (6–10%), renal failure requiring dialysis (25–35%) and local wound infection (up to 37%) [6,7,9,11,12]. Despite these risks, long-term survival is acceptable for those surviving the first 30 days post-transplant, with 1-year survival rates exceeding 80% in selected populations [6]. Early post-transplant mortality remains concentrated in the first month, underscoring the importance of perioperative management and risk mitigation.

## 4. Conclusions

A shorter duration of ECMO support prior to heart transplantation, avoidance of mechanical ventilation and renal insufficiency, and consideration of bridging to LVAD when feasible are associated with improved patient outcomes. Ongoing research is needed to further define optimal timing and patient selection, as well as to reduce perioperative complications in this high-risk population.

## Data Availability

The original data presented in the study are included in the article, further inquiries can be directed to the corre-sponding author.

## Supplementary Materials

The following supporting information can be downloaded at: https://www.mdpi.com/article/10.3390/reports9020105/s1, File S1: Supplementary material providing a detailed description of the sensoring system and the LANDING ADVANCE monitoring platform used in the ECMOLIFE system, including technical specifications and measured parameters.

## Abbreviations

The following abbreviations are used in this manuscript:

ACT	Activated Clotting Time.
ASA	Acetylsalicylic Acid.
BP	Blood Pressure.
CT	Common Trunk.
CX	Circumflex Coronary Artery.
DES	Drug-Eluting Stent.
ECMO	Extracorporeal Membrane Oxygenation.
EF	Ejection Fraction.
ICU	Intensive Care Unit.
IV	Intravenous.
LAD	Left Anterior Descending Coronary Artery.
LVAD	Left Ventricular Assist Device.
PCI	Percutaneous Coronary Intervention.
PC	Phosphorylcholine.
POT	Proximal Optimization Technique.
PTCA	Percutaneous Transluminal Coronary Angioplasty.
SpO_2_	Peripheral Oxygen Saturation.
STEMI	ST-Elevation Myocardial Infarction.
TIMI	Thrombolysis In Myocardial Infarction.
VA ECMO	Veno-Arterial Extracorporeal Membrane Oxygenation.
