# Supplementary material for: ECMO Before Heart Transplantation: Early Implantation and Optimized Assistance with the Eurosets ECMOLIFE System and Landing Advance—A Case Report
Source: Reports (MDPI). 2026 Mar 28;9(2):105. doi: 10.3390/reports9020105 (PMC13108209; doi:10.3390/reports9020105)

ECMOLIFE

NON INVASIVE SENSORING SYSTEM FOR OPTIMAL PATIENT MONITORING

The ECMOLIFE system is equipped with 5 non invasive sensors ensuring continuous monitoring of 11 parameters displayed simultaneously and in real time, providing the operator with **full control of the procedure**.

11 Parameters displayed

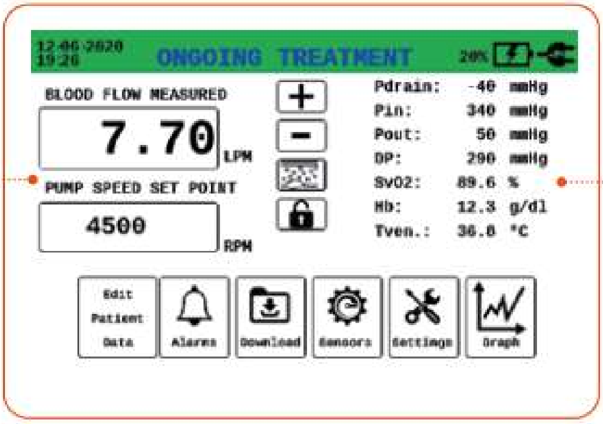

1. Blood flow (LPM)
2. Pump speed (RPM)
3. Drainage pressure (P_{DRAIN})
4. Pre oxygenator pressure (P_{IN})
5. Post oxygenator pressure (P_{OUT})
6. Oxygenator Pressure drop
7. Oxygen venous saturation (SVO_2)
8. Hemoglobin (HB)
9. Blood venous temperature (T_{VEN})
10. Main bubble detector
11. Secondary bubble detector

ECMOLife circuit can be connected to the LANDING ADVANCE.

LANDING ADVANCE displays 20 parameters in real time for optimal patient monitoring.
Thanks to these 20 parameters, HCPs can easily evaluate:

- Procedure efficacy
- Metabolic-hemodynamic patient's pattern
- Interdependency between the Membrane Lung and the Native Lung

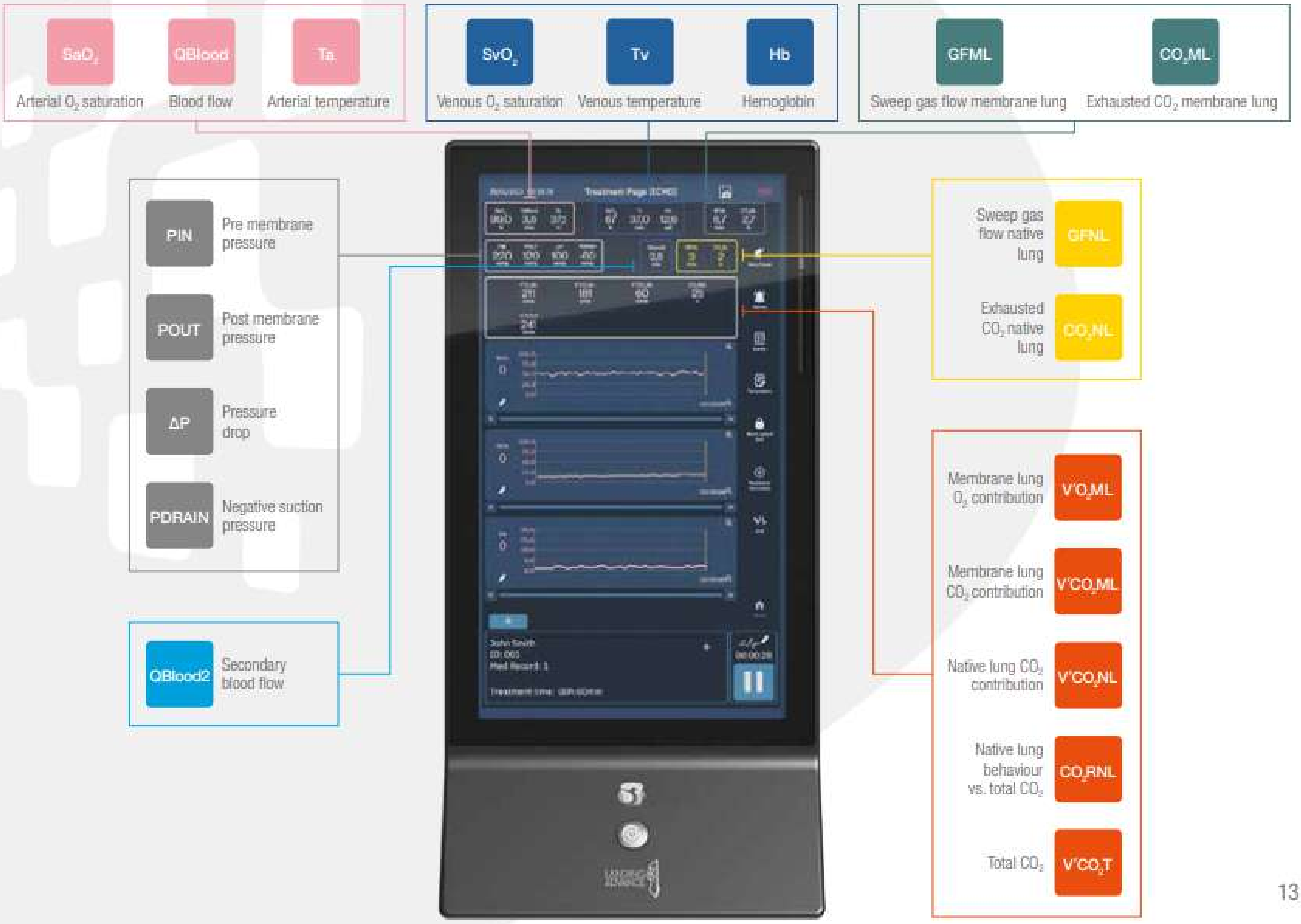

Supplement: Supplementary file 1 [file reports-09-00105-s001.zip › reports-4179487-supplementary.pdf]
